# Sex Differences in Clinical Profile, Revascularization and In-Hospital Outcomes in Patients with ST-Elevation Myocardial Infarction Undergoing Primary Percutaneous Coronary Intervention

**DOI:** 10.3390/jcm15124604

**Published:** 2026-06-13

**Authors:** Corina Cinezan, Camelia Bianca Rus, Timea Claudia Ghitea

**Affiliations:** 1 Department of Medical Disciplines, Faculty of Medicine and Pharmacy, University of Oradea, 410073 Oradea, Romania; rus.cameliabianca@student.uoradea.ro; 2 Clinical County Emergency Hospital Bihor, 410169 Oradea, Romania; 3 Doctoral School of Biological and Biomedical Sciences, University of Oradea, 410087 Oradea, Romania; 4 Pharmacy Department, Faculty of Medicine and Pharmacy, University of Oradea, 410073 Oradea, Romania; timea.ghitea@csud.uoradea.ro

**Keywords:** ST-elevation myocardial infarction, primary percutaneous coronary intervention, sex differences, in-hospital mortality, left ventricular ejection fraction, renal function, ischemic mitral regurgitation, risk stratification, acute coronary syndrome

## Abstract

**Background/Objectives**: Sex differences in ST-elevation myocardial infarction (STEMI) outcomes persist despite advances in primary percutaneous coronary intervention (PCI), but whether female sex independently influences early mortality remains unclear. study aimed to assess sex-based differences in clinical characteristics, management, in-hospital outcomes and to determine whether female sex independently predicts in-hospital mortality. **Methods**: This retrospective observational study included 512 consecutive patients with STEMI presenting within 6 h of symptom onset and treated with primary PCI. Clinical, laboratory, echocardiographic and angiographic data were analyzed. The primary endpoint was in-hospital mortality. Multivariable logistic regression identified independent predictors of mortality. **Results**: Women comprised 32.0% of the cohort and were older than men (median 69 vs. 59 years, *p* < 0.001), with a higher prevalence of diabetes and hypertension, but lower rates of smoking (all *p* < 0.001). Women had lower hemoglobin levels and a higher prevalence of moderate-to-severe mitral regurgitation (17.1% vs. 8.0%, *p* = 0.004). Procedural characteristics, including door-to-balloon time and complete revascularization, were similar between sexes. Crude in-hospital mortality was higher in women (13.4% vs. 7.5%, *p* = 0.047); however, female sex was not independently associated with mortality after adjustment (adjusted OR 1.07, 95% CI 0.48–2.41; *p* = 0.864). Lower LVEF and reduced GFR were the strongest independent predictors of death. **Conclusions**: Higher mortality in women is primarily driven by a more adverse clinical profile rather than sex itself, emphasizing the importance of early risk stratification and management.

## 1. Introduction

Sex differences in cardiovascular disease have been increasingly recognized as an important determinant of clinical presentation, management and outcomes [1,2]. In the context of STEMI, women have consistently been reported to experience worse outcomes compared with men, including higher in-hospital and short-term mortality [3,4,5,6]. However, the underlying mechanisms driving these disparities remain incompletely understood [2,7].

Several factors have been proposed to explain the excess risk observed in women. Women with STEMI tend to present at an older age and have a higher burden of comorbidities, such as hypertension and diabetes mellitus [3,5,8]. In addition, atypical symptom presentation and delays in seeking medical care have been reported more frequently in women, potentially leading to delayed reperfusion [9,10]. Historical data have also suggested sex-based differences in treatment strategies, including lower rates of invasive management and longer door-to-balloon times in women [6,11].

In the contemporary era of primary percutaneous coronary intervention, with standardized STEMI networks and improved adherence to guideline-directed therapy, some of these disparities may have diminished [12,13]. Nevertheless, whether female sex independently contributes to worse outcomes or simply reflects differences in baseline risk profile remains a matter of ongoing debate. Furthermore, limited data are available regarding sex-specific predictors of mortality and the role of factors such as left ventricular dysfunction, renal impairment and ischemic mitral regurgitation in this context [14,15].

Understanding whether outcome differences are attributable to biological sex or to associated clinical characteristics has important implications for risk stratification and management strategies. Identifying sex-specific predictors of adverse outcomes may also contribute to more individualized care and improved prognostication.

Therefore, the aim of the present study was to evaluate sex differences in clinical characteristics, revascularization patterns and in-hospital outcomes among patients presenting with STEMI treated with primary PCI within 6 h of symptom onset. In addition, we sought to determine whether female sex is an independent predictor of in-hospital mortality and to explore sex-specific determinants of adverse outcomes.

## 2. Materials and Methods

### 2.1. Study Population

This retrospective observational study included 512 consecutive patients admitted with STEMI and treated with primary PCI within 6 h of symptom onset, at the Cardiology Department of the Clinical Emergency County Hospital, Oradea, Romania, between January 2021 and August 2025. STEMI was defined according to contemporaneous guideline criteria, based on the presence of ischemic symptoms, persistent ST-segment elevation, new left bundle branch block and/or presumed new ischemic electrocardiographic changes, accompanied by elevated cardiac biomarkers.

All data were obtained from institutional electronic medical records and catheterization laboratory databases.

### 2.2. Inclusion Criteria

Patients were included if they met the following criteria: diagnosis of STEMI according to current guideline definitions, presentation within 6 h of symptom onset, treatment with primary PCI and availability of complete data for the analysis. Restricting inclusion to patients presenting within 6 h of symptom onset was intended to reduce heterogeneity related to delayed reperfusion and infarct evolution, thereby allowing more reliable assessment of sex-related differences in clinical profile and early outcomes. We acknowledge that this selection criterion may have introduced some degree of selection bias by excluding patients with delayed presentation, who may have a different clinical risk profile and potentially worse outcomes. However, restricting the cohort to patients presenting within 6 h was intended to minimize variability related to infarct evolution and delayed reperfusion, thereby allowing a more standardized assessment of sex-related differences in early outcomes following primary PCI.

### 2.3. Exclusion Criteria

Patients were excluded if they had: non-ST elevation myocardial infarction, heart failure or prior significant valvular heart disease, missing key clinical or outcome data and presentation beyond 6 h of symptoms onset.

### 2.4. Clinical and Laboratory Evaluation

Baseline demographic data, cardiovascular risk factors (hypertension, diabetes mellitus, smoking status) and laboratory parameters, including lipid profile, hemoglobin levels, renal function (GFR) and hematological indices, were collected at admission. Hypertension was defined as a prior diagnosis or use of antihypertensive medication. Diabetes mellitus was defined as a prior diagnosis or use of glucose-lowering therapy. Current smoking was defined as active tobacco use at the time of admission. Obesity was defined according to standard body mass index criteria. Dyslipidemia was defined as LDL cholesterol >130 mg/dL or use of lipid-lowering therapy. Glomerular filtration rate (GFR) was estimated using standard equations based on serum creatinine. In-hospital outcomes including mortality were recorded.

### 2.5. Echocardiographic Assessment

Transthoracic echocardiography was performed during hospitalization according to standard practice. Left ventricular ejection fraction (LVEF) was assessed using the Simpson biplane method. Moderate-to-severe ischemic mitral regurgitation (MR) was identified and recorded.

### 2.6. Angiographic and Procedural Assessment

All patients underwent primary percutaneous coronary intervention of the infarct-related artery. Door-to-balloon time was defined as the interval from hospital arrival to first balloon inflation during primary PCI. Complete revascularization was defined as successful treatment of all significant coronary lesions during the index hospitalization. The decision regarding the revascularization strategy, including treatment of non-culprit lesions, was made by the interventional cardiologist according to clinical and angiographic characteristics. After primary PCI, all patients received medical treatment according to current guidelines.

### 2.7. Statistical Analysis

Continuous variables were expressed as median (interquartile range) or mean ± standard deviation as appropriate and compared using Student’s *t*-test or Mann–Whitney U test. Categorical variables were expressed as counts and percentages and compared using the chi-square test or Fisher’s exact test. Multivariable logistic regression analysis was performed to identify independent predictors of in-hospital mortality. Variables included in the model were selected based on clinical relevance and univariate significance. Adjusted odds ratios (ORs) with 95% confidence intervals (CIs) were reported. Sex-stratified analyses were also performed to explore predictors of mortality separately in men and women. A *p*-value < 0.05 was considered statistically significant. Statistical analyses were performed using standard statistical software.

Given the limited number of in-hospital mortality events, a sensitivity analysis was performed using a parsimonious multivariable logistic regression model including sex, age, left ventricular ejection fraction and glomerular filtration rate. Sex-stratified analyses were considered exploratory because of the limited number of mortality events within each subgroup.

## 3. Results

### 3.1. Baseline Clinical Characteristics by Sex

Among 512 patients, 164 (32.0%) were women and 348 (68.0%) were men. Women were significantly older than men. Women more frequently had diabetes mellitus and hypertension, whereas current smoking was substantially more common in men. No significant sex differences were observed for obesity or dyslipidemia.

Baseline clinical characteristics of the study population according to sex are summarized in Table 1, highlighting differences in age, cardiovascular risk factors and comorbidities between women and men.

### 3.2. Clinical Presentation, Laboratory Profile and Management

Infarct territory did not differ significantly by sex and left ventricular ejection fraction was similar in women and men. However, women had a higher prevalence of moderate-to-severe MR. Women also had lower hemoglobin levels, higher creatinine levels, higher RDW and higher platelet counts. GFR, white blood cell indices, mean platelet volume and door-to-balloon time were similar between sexes; median door-to-balloon time was 95 min in women and 89.5 min in men. Complete revascularization was numerically less frequent in women than in men, but the difference was not statistically significant.

Clinical presentation, laboratory findings, procedural characteristics and in-hospital outcomes stratified by sex are presented in Table 2, allowing comparison of disease severity, management and early outcomes between women and men.

### 3.3. In-Hospital Mortality and Regression Analysis

Crude in-hospital mortality was higher in women than in men. In univariable analysis, female sex, older age, diabetes, moderate-to-severe MR, higher RDW, lower hemoglobin levels, lower GFR, lower ejection fraction and incomplete revascularization were associated with death. The strongest crude associations were observed for lower ejection fraction, lower GFR, moderate-to-severe MR and complete revascularization.

The results of the univariable logistic regression analysis identifying predictors of in-hospital mortality in the overall cohort are shown in Table 3.

Multivariable logistic regression models were constructed to identify independent predictors of in-hospital mortality. Variables included in the models were selected based on clinical relevance and/or statistical significance in univariable analysis. In sex-stratified multivariable models, higher ejection fraction and higher GFR remained independently protective in both sexes, whereas moderate-to-severe MR was independently associated with mortality only in men. In women, complete revascularization showed a protective trend, although it did not reach statistical significance.

Multivariable logistic regression analyses of independent predictors of in-hospital mortality in the overall cohort and in sex-stratified models are presented in Table 4.

### 3.4. Sensitivity Analysis

Given the limited number of mortality events, an additional parsimonious multivariable logistic regression model was constructed including only sex, age, left ventricular ejection fraction and glomerular filtration rate. In this sensitivity analysis, female sex was not significantly associated with in-hospital mortality (OR 1.25, 95% CI 0.60–2.64; *p* = 0.552). Lower left ventricular ejection fraction (OR 0.91 per 1% increase, 95% CI 0.88–0.94; *p* < 0.001) and lower glomerular filtration rate (OR 0.96 per 1 mL/min/1.73 m^2^ increase, 95% CI 0.95–0.98; *p* < 0.001) remained independently associated with mortality. These findings were consistent with the primary multivariable analysis, support the robustness of the main results and are illustrated in Table 5.

**Figure 1:**
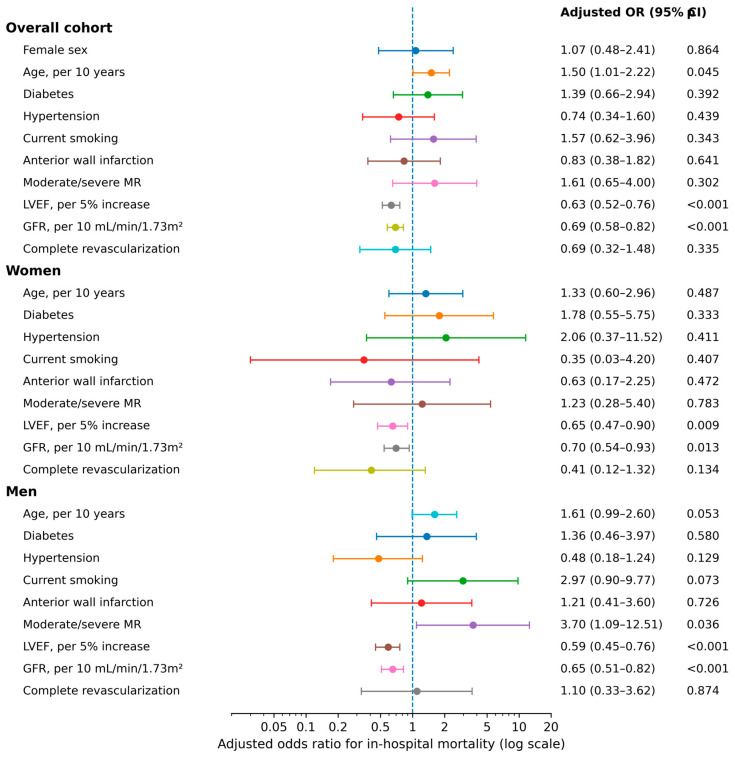
Adjusted predictors of in-hospital mortality in the overall cohort and in sex-stratified models. Points indicate adjusted odds ratios and horizontal lines indicate 95% confidence intervals. The dashed vertical line marks OR = 1. Models were adjusted for clinically relevant covariates including age, diabetes, hypertension, smoking status, infarct location, mitral regurgitation, left ventricular ejection fraction, renal function and completeness of revascularization.

Figure 1 illustrates the adjusted predictors of in-hospital mortality derived from multivariable logistic regression analyses in the overall cohort and in sex-stratified models, including adjusted odds ratios and 95% confidence intervals.

## 4. Discussion

In this cohort of STEMI patients undergoing primary PCI within 6 h of presentation, we observed significant sex-related differences in baseline characteristics, clinical profile and unadjusted outcomes. Women presented at an older age and with a higher burden of comorbidities, including diabetes and hypertension, while men were more frequently smokers. Importantly, women also exhibited a higher prevalence of moderate-to-severe mitral regurgitation and less favorable hematological parameters at admission. Although crude in-hospital mortality was significantly higher in women, sex was not an independent predictor of mortality after adjustment for key clinical variables, suggesting that the observed disparity is largely mediated by differences in baseline risk profile rather than sex itself.

### 4.1. Sex Differences in Clinical Presentation

Consistent with prior studies, women in our cohort were nearly a decade older than men at presentation and had a higher prevalence of cardiometabolic risk factors. This aligns with the well-described phenomenon of delayed manifestation of coronary artery disease in women, likely related to hormonal protection earlier in life [1,4]. The lower prevalence of smoking among women reflects population-level trends but does not offset their higher burden of other risk factors. The higher rate of moderate-to-severe mitral regurgitation observed in women is noteworthy. This may reflect more advanced ischemic injury at presentation or differences in myocardial remodeling and microvascular dysfunction [2]. Additionally, women had lower hemoglobin levels and worse renal function markers, both of which are known to adversely influence outcomes in acute coronary syndromes [16].

### 4.2. Revascularization and Procedural Characteristics

In contrast to some prior reports, we did not observe significant sex differences in door-to-balloon time or rates of complete revascularization. This suggests that, within our center, contemporary STEMI care pathways may have mitigated historical disparities in access to timely reperfusion therapy. The absence of differences in procedural metrics strengthens the interpretation that outcome disparities are not driven by treatment delays but rather by underlying patient characteristics. This finding is particularly relevant in the context of ongoing efforts to standardize STEMI care and reduce sex-based inequities.

### 4.3. Outcomes and Mortality

Women had nearly double the crude in-hospital mortality compared to men. However, this association was no longer significant after adjustment for age, left ventricular ejection fraction and renal function. These findings suggest that female sex was not statistically independently associated with in-hospital mortality in this cohort. However, the confidence interval surrounding the adjusted estimate remains relatively wide, indicating that a clinically meaningful association cannot be completely excluded. Instead, reduced ejection fraction and impaired renal function emerged as the strongest predictors of in-hospital mortality in both sexes. These variables likely reflect the extent of myocardial injury and systemic vulnerability at presentation. Interestingly, moderate-to-severe mitral regurgitation was an independent predictor of mortality in men but not in women. While this may be due to differences in sample size or statistical power, it could also indicate sex-specific pathophysiological responses to ischemic injury that warrant further investigation.

The strong prognostic value of left ventricular ejection fraction is biologically plausible because it reflects the degree of myocardial damage and residual ventricular function following infarction. Similarly, impaired renal function represents a marker of systemic vascular disease, greater comorbidity burden and increased susceptibility to ischemic and procedural complications. The combination of ventricular dysfunction and renal impairment has consistently been associated with adverse outcomes after STEMI and constitutes a major component of several established risk prediction models.

### 4.4. Clinical Implications

Our findings highlight that the higher mortality observed in women with STEMI is largely attributable to their older age and more adverse clinical profile at presentation rather than differences in treatment delivery. This underscores the importance of: earlier detection and aggressive management of cardiovascular risk factors in women, heightened awareness of atypical or delayed presentations and careful risk stratification incorporating renal function and left ventricular performance. Moreover, the lack of sex differences in procedural timelines suggests that standardized STEMI networks can effectively reduce disparities in acute care delivery.

Because the present study focused exclusively on in-hospital outcomes, conclusions regarding long-term prognosis cannot be drawn. Previous studies have suggested that sex-related differences may persist beyond hospitalization, particularly with respect to recurrent cardiovascular events, heart failure development and long-term mortality. Further studies with extended follow-up are therefore needed to determine whether the present findings remain consistent over time.

### 4.5. Comparison with Existing Literature

Our findings are consistent with previous studies showing that women with STEMI undergoing primary PCI are generally older and present with a higher burden of comorbidities, including diabetes and hypertension, while smoking remains more prevalent among men. These sex-related differences have been consistently reported in large registries such as the National Registry of Myocardial Infarction (NRMI), where women had higher crude mortality, largely attributable to older age and a less favorable baseline clinical profile [4]. Similar observations were reported in the GRACE registry, which demonstrated that women with acute coronary syndromes were older and had more comorbidities at presentation [17].

Data from contemporary registries, including SWEDEHEART, ACTION Registry-GWTG and the FAST-MI registry, demonstrated higher unadjusted mortality in women with STEMI; however, this difference was significantly attenuated after adjustment for age, comorbidities and treatment-related factors [18,19,20]. Our results align with these observations, as female sex was associated with higher crude in-hospital mortality but was not an independent predictor after multivariable adjustment.

A meta-analysis by [21] involving patients treated with primary PCI also reported higher mortality rates in women, largely explained by delayed presentation and greater baseline risk burden rather than sex alone. Similar findings were reported by [22], who demonstrated that sex differences in mortality were markedly reduced after risk adjustment. In addition, [7] showed that women with STEMI continue to experience worse outcomes, particularly when presenting later and with more advanced clinical severity.

Our finding that reduced LVEF and impaired renal function were the strongest independent predictors of in-hospital mortality is consistent with prior evidence identifying these variables as major prognostic markers after STEMI. These factors are incorporated into established risk models such as the CADILLAC score [23,24].

Unlike earlier reports describing lower rates of invasive treatment in women, we found no significant sex differences in procedural characteristics, including door-to-balloon time and complete revascularization, which may reflect improvements in contemporary STEMI care [25]. Overall, our findings support the notion that worse outcomes in women are primarily driven by higher baseline risk rather than female sex itself.

Recent evidence from the PRAISE registry further emphasizes the importance of the baseline clinical profile and contemporary management strategies in determining prognosis after acute coronary syndromes. The registry demonstrated that differences in outcomes are strongly influenced by patient characteristics and comorbidity burden, supporting our observation that crude mortality differences between women and men are largely explained by differences in baseline risk profile rather than sex alone [26].

Nevertheless, not all contemporary studies have reported complete attenuation of sex-related differences after adjustment. Several registries continue to demonstrate disparities in presentation patterns, treatment delays and long-term outcomes among women with STEMI. These findings suggest that sex-related differences are multifactorial and may reflect the combined influence of biological, clinical and healthcare-system factors. Our results support the concept that baseline clinical risk profile is a major contributor to early mortality differences following primary PCI. They should be interpreted within the context of a single-center retrospective study and should not be considered definitive evidence that sex-related differences have been completely eliminated in contemporary STEMI care.

### 4.6. Strengths

This study has several important strengths. First, it includes a relatively large, contemporary cohort of STEMI patients (*n* = 512) treated exclusively with primary PCI within the first 6 h of presentation, ensuring a clinically homogeneous population and reflecting real-world practice in the modern reperfusion era. Second, the dataset is comprehensive and integrates detailed clinical, laboratory and procedural variables, including renal function, hematological parameters, left ventricular ejection fraction and the presence of moderate-to-severe mitral regurgitation. This allowed for a robust and clinically meaningful multivariable analysis of predictors of in-hospital mortality. Third, the study specifically explores sex differences using both overall and sex-stratified analyses. This approach provides additional insight into whether predictors of mortality differ between women and men, moving beyond simple comparisons of outcomes and contributing to a more nuanced understanding of sex-based disparities. Fourth, the inclusion of procedural variables such as door-to-balloon time and completeness of revascularization enabled assessment of potential treatment-related disparities. The finding of no significant differences in these parameters strengthens the conclusion that outcome differences are primarily driven by baseline risk rather than inequities in acute care delivery. Fifth, all patients were managed within a single center using standardized protocols for STEMI care, which reduces variability in treatment strategies and enhances internal validity. Finally, the study addresses a clinically relevant and ongoing question regarding sex disparities in STEMI outcomes, providing data that are directly applicable to everyday clinical practice and supporting efforts toward more personalized and equitable cardiovascular care.

### 4.7. Limitations

This study has several limitations that should be acknowledged. First, it is a single-center observational study, which may limit the generalizability of the findings to other populations or healthcare systems with different patient characteristics or treatment protocols. Second, although the overall sample size was moderate, the number of in-hospital mortality events was relatively small; only 48 in-hospital deaths occurred. Consequently, the number of events available for multivariable modelling was limited, particularly in the sex-stratified analyses. Therefore, these subgroup analyses should be considered exploratory and interpreted with caution because of the potential risk of model overfitting and reduced statistical power. Third, residual confounding cannot be excluded. Despite adjustment for key clinical variables such as age, ejection fraction, renal function and mitral regurgitation, unmeasured factors, such as infarct size, microvascular obstruction, extent of myocardial salvage or differences in pharmacological therapy, may have influenced outcomes. Fourth, the study focuses exclusively on in-hospital outcomes and does not include short- or long-term follow-up. Therefore, potential sex differences in post-discharge mortality, heart failure development, or recurrent ischemic events could not be assessed. Fifth, some variables were assessed at a single time point on admission, which may not fully capture dynamic changes during hospitalization. In addition, echocardiographic parameters such as ejection fraction and mitral regurgitation were not evaluated by a core laboratory, which may introduce measurement variability. Sixth, although procedural metrics such as door-to-balloon time and completeness of revascularization were included, more granular procedural details, such as lesion complexity, use of adjunctive devices, or microvascular perfusion indices, were not available and could provide further insights into sex-related differences.

Seventh, information regarding socioeconomic status, educational level, pre-hospital management, emergency medical service utilization and delays in seeking medical attention before hospital arrival was not available in the present dataset. These factors may influence outcomes and could contribute to residual confounding.

Furthermore, detailed information regarding pharmacological treatment before admission, during hospitalization and at discharge was not systematically available for analysis. Therefore, the potential impact of sex-related differences in medical therapy on clinical outcomes could not be evaluated.

Finally, because this was a retrospective observational study based on consecutive eligible patients, no formal a priori sample size calculation was performed. Although the overall cohort included 512 patients, the number of mortality events was relatively limited. Consequently, the study may have been underpowered to detect modest independent associations, particularly in sex-specific analyses.

## 5. Conclusions

In STEMI patients treated with primary PCI, women exhibited higher crude in-hospital mortality; however, this difference was largely explained by older age and a higher burden of comorbidities. Female sex was not statistically independently associated with in-hospital mortality after adjustment. However, the confidence interval around the adjusted estimate suggests that a clinically meaningful association cannot be completely excluded. Left ventricular dysfunction and renal impairment were the strongest determinants of adverse outcomes in both sexes. These findings emphasize the need for improved primary prevention and early risk recognition in women, rather than differences in acute procedural management.

## Figures and Tables

**Table 1:** Baseline clinical characteristics according to sex.

Variable	Women (*n* = 164)	Men (*n* = 348)	*p*-Value
Age, years	69.0 (62.0–75.0)	59.0 (50.0–69.0)	<0.001
Diabetes mellitus	63 (38.4%)	77 (22.1%)	<0.001
Hypertension	125 (76.2%)	215 (61.8%)	0.002
Current smoking	38 (23.2%)	175 (50.3%)	<0.001
Obesity	18 (11.0%)	31 (8.9%)	0.561
Dyslipidemia (LDL > 130 mg/dL)	56 (34.1%)	106 (30.5%)	0.462

Note: Values are presented as median (interquartile range [IQR]) for continuous variables and as counts (percentages) for categorical variables. Comparisons between women and men were performed using Student’s *t*-test or Mann–Whitney U test for continuous variables, as appropriate and the chi-square test or Fisher’s exact test for categorical variables.

**Table 2:** Clinical presentation, laboratory profile, treatment and in-hospital outcomes in patients with STEMI according to sex.

Variable	Women (*n* = 164)	Men (*n* = 348)	*p*-Value
Anterior wall infarction	72 (43.9%)	165 (47.4%)	0.517
Inferior wall infarction	89 (54.3%)	179 (51.4%)	0.614
Lateral wall infarction	19 (11.6%)	43 (12.4%)	0.917
Posterior wall infarction	29 (17.7%)	66 (19.0%)	0.821
Right ventricular involvement	14 (8.5%)	33 (9.5%)	0.856
Ejection fraction, %	45 (36–50)	45 (40–50)	0.836
Moderate-to-severe mitral regurgitation	28 (17.1%)	28 (8.0%)	0.004
Hemoglobin, g/dL	13.3 (12.0–14.4)	14.9 (13.9–15.8)	<0.001
Creatinine, mg/dL	1 (0.8–1.3)	0.9 (0.8–1.1)	<0.001
GFR, mL/min/1.73 m^2^	70.5 (50.0–90.0)	76.0 (56.0–92.0)	0.247
Door-to-balloon time, min	95 (65–144)	90 (62–140)	0.351
WBC, ×10^3^/µL	11.0 (8.8–14.8)	11.7 (9.4–14.3)	0.287
Neutrophils, ×10^3^/µL	7.8 (5.9–11.3)	8.6 (6.1–11.0)	0.384
Lymphocytes, ×10^3^/µL	1.8 (1.2–2.8)	1.8 (1.3–2.8)	0.727
RDW, %	13.6 (13.0–14.3)	13.3 (12.8–14.1)	0.012
MPV, fL	10.6 (10.0–11.4)	10.6 (10.1–11.3)	0.998
Platelets, ×10^3^/µL	246 (209–302)	234 (199–270)	0.009
Complete revascularization	117 (71.3%)	271 (77.9%)	0.134
In-hospital mortality	22 (13.4%)	26 (7.5%)	0.047

Note: Continuous variables are expressed as median (IQR) and categorical variables as counts (percentages). Between-group comparisons were performed using Student’s *t*-test or Mann–Whitney U test for continuous variables and the chi-square test or Fisher’s exact test for categorical variables.

**Table 3:** Univariable logistic regression for in-hospital mortality in the overall cohort.

Predictor	Odds Ratio (95% CI)	*p*-Value
Female sex	1.92 (1.05–3.50)	0.034
Age (per 10 years)	2.32 (1.71–3.15)	<0.001
Diabetes	2.25 (1.23–4.14)	0.009
Hypertension	0.91 (0.49–1.70)	0.779
Current smoking	0.34 (0.16–0.70)	0.003
Anterior wall infarction	1.56 (0.85–2.83)	0.148
Moderate-to-severe MR	4.14 (2.06–8.32)	<0.001
Complete revascularization	0.37 (0.20–0.68)	0.001
Ejection fraction (per 5%)	0.57 (0.48–0.67)	<0.001
GFR (per 10 mL/min/1.73 m^2^)	0.65 (0.57–0.74)	<0.001
Hemoglobin (per 1 g/dL)	0.66 (0.56–0.78)	<0.001
RDW (per 1%)	1.37 (1.07–1.76)	0.011

Note: Odds ratios (ORs) with 95% confidence intervals (CIs) are presented for each variable. Univariable logistic regression was used to evaluate the association between clinical, laboratory and procedural variables and in-hospital mortality. Continuous variables were analyzed as continuous predictors. Odds ratios for age and GFR are expressed per 10-unit increase, whereas odds ratios for left ventricular ejection fraction are expressed per 5% increase. Odds ratios for hemoglobin and RDW are expressed per 1-unit increase. A two-sided *p*-value < 0.05 was considered statistically significant.

**Table 4:** Multivariable logistic regression analysis of predictors of in-hospital mortality in the overall cohort and stratified by sex.

Predictor	Overall Cohort, Adjusted OR (95% CI)	Women, Adjusted OR (95% CI)	Men, Adjusted OR (95% CI)
Female sex	1.07 (0.48–2.41); *p* = 0.864	—	—
Age (per 10 years)	1.50 (1.01–2.22); *p* = 0.045	1.33 (0.60–2.96); *p* = 0.487	1.61 (0.99–2.60); *p* = 0.053
Diabetes	1.39 (0.66–2.94); *p* = 0.392	1.78 (0.55–5.75); *p* = 0.333	1.36 (0.46–3.97); *p* = 0.580
Hypertension	0.74 (0.34–1.60); *p* = 0.439	2.06 (0.37–11.52); *p* = 0.411	0.48 (0.18–1.24); *p* = 0.129
Current smoking	1.57 (0.62–3.96); *p* = 0.343	0.35 (0.03–4.20); *p* = 0.407	2.97 (0.90–9.77); *p* = 0.073
Anterior wall infarction	0.83 (0.38–1.82); *p* = 0.641	0.63 (0.17–2.25); *p* = 0.472	1.21 (0.41–3.60); *p* = 0.726
Moderate-to-severe MR	1.61 (0.65–4.00); *p* = 0.302	1.23 (0.28–5.40); *p* = 0.783	3.70 (1.09–12.51); *p* = 0.036
Ejection fraction (per 5%)	0.63 (0.52–0.76); *p* < 0.001	0.65 (0.47–0.90); *p* = 0.009	0.59 (0.45–0.76); *p* < 0.001
GFR (per 10 mL/min/1.73 m^2^)	0.69 (0.58–0.82); *p* < 0.001	0.70 (0.54–0.93); *p* = 0.013	0.65 (0.51–0.82); *p* < 0.001
Complete revascularization	0.69 (0.32–1.48); *p* = 0.335	0.41 (0.12–1.32); *p* = 0.134	1.10 (0.33–3.62); *p* = 0.874

Note: Adjusted odds ratios (ORs) with 95% confidence intervals (CIs) are reported. Continuous variables were modeled per clinically meaningful increments: age per 10 years, GFR per 10 mL/min/1.73 m^2^ and left ventricular ejection fraction per 5% increase. Sex-stratified models included the same covariates except sex. A two-sided *p*-value < 0.05 was considered statistically significant.

**Table 5:** Sensitivity analysis using a parsimonious multivariable logistic regression model for in-hospital mortality.

Predictor	Adjusted OR (95% CI)	*p*-Value
Female sex	1.25 (0.60–2.64)	0.552
Age (per year)	1.04 (1.00–1.07)	0.058
Left ventricular ejection fraction (per 1% increase)	0.91 (0.88–0.94)	<0.001
GFR (per 1 mL/min/1.73 m^2^ increase)	0.96 (0.95–0.98)	<0.001

Note: Adjusted odds ratios (ORs) with 95% confidence intervals (CIs) are presented. The sensitivity model included sex, age, left ventricular ejection fraction and glomerular filtration rate. Variables were selected based on clinical relevance and to reduce the risk of model overfitting, given the limited number of in-hospital mortality events. A two-sided *p*-value < 0.05 was considered statistically significant.

## Data Availability

The raw data supporting the conclusions of this article will be made available by the authors on request. The original contributions presented in this study are included in the article. Further inquiries can be directed to the corresponding author.
